# Family accommodation in childhood, adolescent, and young adult cancer survivors: A scoping review protocol

**DOI:** 10.1371/journal.pone.0356107

**Published:** 2026-08-14

**Authors:** Rinat Nissim, Rouhi Fazelzad, Sarah Daniels, Jake Heinbuch, Aditya Lumb, Luke Heinbuch, Andreea A. Manea, Uri Berger

**Affiliations:** 1 Department of Supportive Care, Princess Margaret Cancer Centre, University Health Network, Toronto, Ontario, Canada; 2 Department of Psychiatry, Temerty Faculty of Medicine, University of Toronto, Toronto, Ontario, Canada; 3 Library and Information Service, Princess Margaret Cancer Centre, University Health Network, Toronto, Ontario, Canada; 4 Faculty of Health Sciences, Queen’s University, Kingston, Ontario, Canada; 5 Knowledge Translation Program, St. Michael’s Hospital, Unity Health Toronto, Toronto, Ontario, Canada; 6 The Lighthouse Psychological Services PLLC, New York, New York, United States of America; North South University, BANGLADESH

## Abstract

Long-term survival rates for childhood, adolescent, and young adult cancers have increased substantially, yet survivors often face ongoing physical, psychological, and social challenges. Family dynamics play a key role in survivor adjustment. Family accommodation, defined as behaviour modifications by family members intended to reduce distress, may be one such important factor influencing psychological adjustment. Despite extensive research in mental health populations, the role of family accommodation in the context of childhood, adolescent, and young adult cancers has not been explored. The main objective of this scoping review is to map and synthesize existing evidence on how family accommodation has been conceptualized, measured and linked to the health, health-related behaviors, and mental health of childhood, adolescent, and young adult cancer survivors. Secondary objectives are to examine the relationship between family accommodation and caregiver psychological well-being, and to identify how this construct is measured in this context. This review will include studies of any design that investigate family accommodation among individuals diagnosed with cancer between birth and 39 years of age and/or their family members. Database search will extract eligible articles from Medline ALL, Embase Classic +Embase, Emcare, Cochrane Central Register of Controlled Trials, Cochrane Database of Systematic Reviews, PsycInfo on the OvidSP platform, and Scopus from Elsevier from each database inception to October 2024. The review will follow the JBI methodology for scoping review guidance and will be reported according to PRISMA-ScR. By synthesizing available evidence, this review will provide a foundation for understanding how family accommodation shapes adjustment in cancer survivorship. The findings will inform future research and contribute to family-centered survivorship care. This review was preregistered on the Open Science Framework (OSF). Registration https://doi.org/10.17605/OSF.IO/V7UFK.

## Introduction

Long-term survival rates for childhood, adolescent, and young adult (CAYA) cancers have significantly increased due to advancements in diagnosis and treatment. In Western countries, the five-year survival rate now exceeds 90% [1,2]. However, many CAYA cancer survivors face substantial risks of physical effects from their cancer and its treatment. Up to 96% of survivors may develop severe chronic conditions, including endocrine, cardiovascular, neurocognitive, pulmonary, and reproductive disorders [1,3]. In addition to physical effects, survivors often experience psychological, social, and functional challenges impacting their overall health and quality of life. A recent meta-analysis demonstrated that CAYA cancer survivors are at increased risk of depression and anxiety relative to both matched controls and their siblings [2].

Family systems perspectives emphasize that physical and mental health challenges occur within a network of reciprocal interactions among family members, with shared behaviour patterns influencing overall adjustment outcomes [4]. Family accommodation refers to patterns in which family members, most often parents or primary caregivers, engage in behaviours aimed at preventing, alleviating, or minimizing a child’s distress. These behaviours may include providing excessive reassurance, modifying family routines, or helping the child avoid situations that trigger distress [5]. Although such behaviours are well-intentioned and may provide short-term relief, research among families of CAYA populations, including those with anxiety disorders, autism and sleep difficulties, indicates that higher levels of family accommodation are associated with greater severity of the child’s symptoms, increased functional impairment, and poorer treatment outcomes. [5–7]. Importantly, interventions aimed at reducing family accommodation in CAYA populations have been shown to reduce the child’s symptoms, improve their functioning, and reduce parental burden [8–10].

Although family accommodation has been extensively studied in pediatric mental health contexts, its relevance to physical disease, and cancer in particular, remains underexplored. In the context of CAYA cancers, the high psychosocial distress experienced by families due to cancer and its treatment may potentially lead to family accommodation during the cancer diagnosis phase, treatment phase and/or post-treatment phase, negatively impacting both the CAYA cancer survivor’s adjustment and parental burden [11]. Yet, no synthesis of how family accommodation has been conceptualized, measured, or linked to outcomes in this context has been conducted. Moreover, the distinction between family accommodation and related caregiving constructs, such as parental overprotection, family rules and routines, and dependence-promoting behaviours remains unclear, particularly in the context of CAYA cancers. Understanding and addressing family accommodation in the context of CAYA cancers is crucial for developing effective strategies to enhance adjustment and overall well-being for families navigating CAYA cancers.

The proposed scoping review is a crucial preliminary step in planning and developing an intervention study for CAYA cancer survivors and their families. A preliminary search of MEDLINE, the Cochrane Database of Systematic Reviews, JBI Evidence Synthesis, PROSPERO (International prospective register of systematic reviews), and Open Science Framework (OSF) was conducted, and no current or underway systematic reviews or scoping reviews on the topic were identified.

### Review objectives and questions

Our primary objective is to conduct a literature review to map and synthesize available evidence on how family accommodation has been conceptualized, measured and linked to the health, health-related behaviors, and mental health of CAYA cancer survivors.

Secondary objectives of this scoping review are to identify: 1) how family accommodation is linked to the emotional and psychological well-being of parents or other family caregivers of CAYA cancer survivors; and 2) how family accommodation is measured in the context of cancer.

## Methods

This scoping review will follow the Joanna Briggs Institute (JBI) manual for evidence synthesis for scoping reviews [12], and be reported according to the Preferred Reporting Items for Systematic Reviews and Meta-Analyses extension for scoping reviews (PRISMA ScR; 13). We will employ a scoping review approach rather than a systematic review due to the heterogeneity of study designs, definitions, measurement approaches, and associated outcomes. The review protocol was registered with the Open Science Framework (OSF) (Registration DOI 10.17605/OSF.IO/V7UFK), registration number:. At the time of manuscript re-submission, title and abstract screening had been completed and full-text review was underway. Data extraction and synthesis had not yet begun. Any amendments to this protocol will be documented with their rationale and transparently reported in the final scoping review.

### Inclusion criteria

We used the PEO (Population, Exposure, Outcomes) framework to structure our study inclusion criteria.

Our “Population” of interest for this review will include individuals diagnosed with cancer between birth and 39 years of age and their families. Eligible family members include parents, legal guardians, siblings, spouses or partners, and other family members or informal caregivers identified by study authors as providing care or support to the survivor. Consistent with the National Cancer Institute definition of survivorship [14], studies conducted at any point along the cancer survivorship trajectory, from diagnosis through treatment and post-treatment follow-up, will be included. Studies including mixed-age cancer populations will be eligible only if data for individuals diagnosed between birth and 39 years of age are reported separately or can be extracted independently. Finally, we will exclude studies involving individuals with a terminal cancer diagnosis or those receiving palliative or end-of-life care.

Our review will focus on family accommodation as the “Exposure” of interest. We will include descriptive or intervention studies that evaluate: 1) how family accommodation influences the health, health-related behaviors, and mental health of CAYA cancer survivors; and 2) how family accommodation affects the emotional and psychological well-being of parents or other family caregivers. We will also explore how family accommodation in the context of CAYA cancer survivors is conceptualized and measured. Studies will be eligible if they examine family accommodation in any healthcare, home, or community setting relevant to CAYA cancer survivorship, with family accommodation referring to behaviours such as excessive reassurance, modification of family routines, or taking over tasks the survivor could otherwise perform. We will also include studies that examine conceptually related constructs (e.g., parental overprotection, protective buffering, family rules and routines, caregiving adaptations, and dependence-promoting behaviours). We will exclude studies that focus on general emotional support or medically necessary caregiving.

We will not define specific “Outcomes” to allow for the broadest inclusion of relevant literature. However, we aim to capture outcomes related to child health, health-related behaviors, mental health, emotional well-being of family members, and measures used to assess family accommodation.

### Types of studies

This scoping review will consider both experimental and quasi-experimental study designs including randomized controlled trials, non-randomized controlled trials, before and after studies, and interrupted time-series studies. In addition, analytical observational studies including prospective and retrospective cohort studies, case-control studies and analytical cross-sectional studies will be considered for inclusion. This review will also consider descriptive observational study designs including case series, and descriptive cross-sectional studies for inclusion. Qualitative studies will also be considered, including but not limited to, designs such as phenomenology, grounded theory, ethnography, and qualitative description. Relevant systematic reviews will not be included as evidence sources; however, their reference lists will be screened to identify additional eligible primary studies. Gray literature, case studies and opinion papers will not be considered for inclusion in this scoping review.

### Search strategy

First, an initial limited search of MEDLINE was undertaken to identify articles on the topic. The text words contained in the titles and abstracts of relevant articles, and the index terms used to describe the articles were then used to develop a full search strategy.

In collaboration with the information specialist, an extensive literature search was conducted in Medline ALL (Medline and Medline Epub Ahead of print and In-Process & Other Non-Indexed Citations), Embase Classic +Embase, Emcare, Cochrane Central Register of Controlled Trials, Cochrane Database of Systematic Reviews, PsycInfo all from the OvidSP platform, and Scopus from Elsevier from each database inception to October 2024. Each search strategy included a combination of controlled vocabulary terms and text words, adapting the database-specific search syntax. Where applicable, the search was restricted to human studies and publications in English, and excluded books, conferences, dissertations, and preprints (see Supplementary 1 file for Medline ALL search).

The reference lists of any eligible papers will be hand-searched to identify potentially relevant additional articles. Prior to publication, the literature search will be rerun to identify studies published since the original search (October 2024). Newly identified records will undergo the same process of removing duplicate articles, title and abstract screening, full-text review, and data extraction procedures as described above. Any additional eligible studies identified through the updated search will be incorporated into the final review and documented in the PRISMA flow diagram.

### Selection of sources of evidence

Following the search, all identified citations will be collated and uploaded into Covidence and duplicates removed. Following a pilot test, titles and abstracts will be screened by two or more independent reviewers for assessment against the inclusion criteria for the review. Potentially relevant sources will be retrieved in full.

Prior to full-text review, pairs of reviewers will pilot the screening process on a sample of 20 articles. This calibration step will be repeated until at least 80% inter-rater agreement is achieved. Following this, two or more independent reviewers will assess the full text of all remaining selected citations in detail against the inclusion criteria. Reasons for excluding full-text sources of evidence that do not meet the inclusion criteria will be recorded and reported in the scoping review. Any disagreements that arise between the reviewers at each stage of the selection process will be resolved through discussions with a third reviewer. The results of the search and the study inclusion process will be reported in full in the final scoping review and presented in a PRISMA flow diagram [13].

### Data extraction

Data will be extracted from papers included in the scoping review by two or more independent reviewers using a data extraction tool (See Supplementary 2 file) developed and pilot tested by the reviewers. The data extraction tool will capture specific details, including: study characteristics (e.g., author, year, country, study design, setting); participant characteristics (e.g., survivor age at diagnosis, cancer diagnosis, treatment status, family member type); conceptualization of family accommodation; measures used to assess family accommodation; survivor and caregiver outcomes examined; key findings relevant to the review objectives; and study limitations. The data extraction tool will be modified and revised as necessary during the process of extracting data from each included evidence source. Modifications will be detailed in the scoping review. Any disagreements that arise between the reviewers will be resolved through discussion. Authors of papers will be contacted to request missing or additional data, where required.

### Data synthesis

Data will be synthesized and presented in tables and diagrams, aligned with the review’s primary and secondary objectives, and supported by a narrative summary. No critical appraisal will be undertaken for the studies included in this review.

## Discussion

By systematically mapping how family accommodation has been studied among CAYA cancer survivors and their families, this review will provide insights into behaviors that may either facilitate or hinder adjustment. It will identify conceptual overlaps and distinctions with related constructs, highlight methodological gaps, and suggest directions for future research and intervention development. As a scoping review, this study will map the breadth of available evidence rather than evaluate the quality of included studies or establish causal relationships. Findings may also be limited by the availability of published English-language literature and heterogeneity in how family accommodation has been conceptualized and measured. Nevertheless, identifying how family accommodation has been defined, assessed, and linked to survivor and family outcomes may help inform the development of future family-centered survivorship interventions by identifying potentially modifiable caregiver behaviours and highlighting areas requiring further empirical investigation. Ultimately, understanding family accommodation in this context will contribute to a more nuanced framework for CAYA cancer survivorship care, recognizing that survivors’ adjustment and well-being are embedded within interconnected family systems.

## Supporting information

S1 File This is the medline ALL search. (DOCX)

S2 File This is the data extraction tool. (PDF)

S3 File This is the PRISMA-P checklist. (DOCX)
