# Supplementary material for: Family accommodation in childhood, adolescent, and young adult cancer survivors: A scoping review protocol
Source: PLoS One. 2026 Aug 14;21(8):e0356107. doi: 10.1371/journal.pone.0356107 (PMC13475868; doi:10.1371/journal.pone.0356107)
Supplement: Supplementary file 1 [file pone.0356107.s002.pdf]

Data Extraction Tool
**Citation**
Title
Authors
Year
Country
Journal Vol
Journal Issue
URL / DOI
Key Words
**Study Characteristics**
Research Question
Study Aim(s)
Study Design (e.g., qualitative, quantitative, mixed, SysR, ScR, MA)
Setting / Context (e.g., institutional setting, cultural env.)
**Population**
Survivor Age at Diagnosis
Survivor Age at Study
Sex
Cancer Type
Treatment Status / Survivorship Stage (e.g., diagnosis, active treatment, post-treatment, long-term follow-up)
Sample Size
Family Participants Included (e.g., parent, caregiver, sibling, spouse/partner)
**Family Accommodation (FA)**
Definition / Conceptualization (e.g., routine modification, excessive reassurance, participation in avoidance, symptom management behaviours, parental behavioural adaptation, etc.)
Behaviours Examined (e.g., Providing reassurance, Sleeping with the child, Allowing avoidance of medical procedures, Completing tasks for the child, Excusing school attendance, etc.)
Measurement Approach (e.g., validated measure, adapted measure, qualitative interview, investigator-developed items)
Measurement Tool / Instrument (name of scale if applicable)
**Outcomes**
Survivor Outcomes

Caregiver / Family Outcomes
Direction of Association with FA (e.g., negative, positive)
**Other**
Key Findings
Study Limitations
Reviewer Notes
