# Supplementary material for: Family accommodation in childhood, adolescent, and young adult cancer survivors: A scoping review protocol
Source: PLoS One. 2026 Aug 14;21(8):e0356107. doi: 10.1371/journal.pone.0356107 (PMC13475868; doi:10.1371/journal.pone.0356107)
Supplement: Supplementary file 2 [file pone.0356107.s001.docx]

**Ovid MEDLINE(R) ALL 1946 to October 16, 2024**

| **#** | **Searches** | **Results** | **Type** |  |  |  |
| --- | --- | --- | --- | --- | --- | --- |
|  | | | | | | |
| 1 | exp Neoplasms/ | 4030046 | Advanced |  |  |  |
| 2 | Cancer Survivors/ | 10678 | Advanced |  |  |  |
| 3 | neoplas*.tw,kf. | 472185 | Advanced |  |  |  |
| 4 | paraneoplas*.tw,kf. | 13764 | Advanced |  |  |  |
| 5 | cancer*.tw,kf. | 2457226 | Advanced |  |  |  |
| 6 | tumo?r*.tw,kf. | 2180516 | Advanced |  |  |  |
| 7 | onco*.tw,kf. | 452804 | Advanced |  |  |  |
| 8 | metast*.tw,kf. | 675307 | Advanced |  |  |  |
| 9 | multimetast*.tw,kf. | 54 | Advanced |  |  |  |
| 10 | macrometast*.tw,kf. | 1164 | Advanced |  |  |  |
| 11 | micrometast*.tw,kf. | 7468 | Advanced |  |  |  |
| 12 | malig*.tw,kf. | 740673 | Advanced |  |  |  |
| 13 | aberrant crypt foci.tw,kf. | 1627 | Advanced |  |  |  |
| 14 | acanthoma*.tw,kf. | 775 | Advanced |  |  |  |
| 15 | acrospiroma*.tw,kf. | 91 | Advanced |  |  |  |
| 16 | adamantinom*.tw,kf. | 1328 | Advanced |  |  |  |
| 17 | adenocarc*.tw,kf. | 195398 | Advanced |  |  |  |
| 18 | adenofibrom*.tw,kf. | 607 | Advanced |  |  |  |
| 19 | adenolymphom*.tw,kf. | 481 | Advanced |  |  |  |
| 20 | adenomat*.tw,kf. | 21120 | Advanced |  |  |  |
| 21 | adenomyo*.tw,kf. | 5554 | Advanced |  |  |  |
| 22 | adenosarcom*.tw,kf. | 696 | Advanced |  |  |  |
| 23 | adenosquam*.tw,kf. | 3399 | Advanced |  |  |  |
| 24 | ameloblastom*.tw,kf. | 4181 | Advanced |  |  |  |
| 25 | androblastom*.tw,kf. | 99 | Advanced |  |  |  |
| 26 | angiofibrom*.tw,kf. | 2546 | Advanced |  |  |  |
| 27 | angiokeratom*.tw,kf. | 1140 | Advanced |  |  |  |
| 28 | angiolipom*.tw,kf. | 720 | Advanced |  |  |  |
| 29 | angioma*.tw,kf. | 12276 | Advanced |  |  |  |
| 30 | angiomyolipom*.tw,kf. | 4799 | Advanced |  |  |  |
| 31 | angiomyom*.tw,kf. | 178 | Advanced |  |  |  |
| 32 | angiosarcom*.tw,kf. | 7886 | Advanced |  |  |  |
| 33 | apudoma*.tw,kf. | 302 | Advanced |  |  |  |
| 34 | arrhenoblastom*.tw,kf. | 355 | Advanced |  |  |  |
| 35 | astrocytom*.tw,kf. | 18722 | Advanced |  |  |  |
| 36 | blastom*.tw,kf. | 10600 | Advanced |  |  |  |
| 37 | Bowen*.tw,kf. | 3583 | Advanced |  |  |  |
| 38 | Brenner*.tw,kf. | 1695 | Advanced |  |  |  |
| 39 | Buschke-Lowenstein*.tw,kf. | 316 | Advanced |  |  |  |
| 40 | carcin*.tw,kf. | 992347 | Advanced |  |  |  |
| 41 | cementoma*.tw,kf. | 225 | Advanced |  |  |  |
| 42 | chemodectomas*.tw,kf. | 314 | Advanced |  |  |  |
| 43 | cholangiocarcin*.tw,kf. | 20324 | Advanced |  |  |  |
| 44 | chondroblastom*.tw,kf. | 1225 | Advanced |  |  |  |
| 45 | chondroma*.tw,kf. | 4809 | Advanced |  |  |  |
| 46 | chordoma*.tw,kf. | 5032 | Advanced |  |  |  |
| 47 | chondrosarcom*.tw,kf. | 9594 | Advanced |  |  |  |
| 48 | choriocarcin*.tw,kf. | 7722 | Advanced |  |  |  |
| 49 | craniopharyngioma*.tw,kf. | 5381 | Advanced |  |  |  |
| 50 | cystadenofibrom*.tw,kf. | 240 | Advanced |  |  |  |
| 51 | cystosarcom*.tw,kf. | 651 | Advanced |  |  |  |
| 52 | cytoma*.tw,kf. | 403 | Advanced |  |  |  |
| 53 | dermatofibrosarcom*.tw,kf. | 2443 | Advanced |  |  |  |
| 54 | desmoplas*.tw,kf. | 6177 | Advanced |  |  |  |
| 55 | dysgerminoma*.tw,kf. | 1544 | Advanced |  |  |  |
| 56 | DCIS.tw,kf. | 6250 | Advanced |  |  |  |
| 57 | DSRCT.tw,kf. | 444 | Advanced |  |  |  |
| 58 | ependymom*.tw,kf. | 6462 | Advanced |  |  |  |
| 59 | Ewing*.tw,kf. | 12023 | Advanced |  |  |  |
| 60 | fibroadenom*.tw,kf. | 4414 | Advanced |  |  |  |
| 61 | fibroepithelial*.tw,kf. | 1380 | Advanced |  |  |  |
| 62 | fibroma*.tw,kf. | 14887 | Advanced |  |  |  |
| 63 | fibrosarcom*.tw,kf. | 12795 | Advanced |  |  |  |
| 64 | FAMMM.tw,kf. | 88 | Advanced |  |  |  |
| 65 | gangliogliom*.tw,kf. | 1669 | Advanced |  |  |  |
| 66 | ganglioneurom*.tw,kf. | 2514 | Advanced |  |  |  |
| 67 | gastrinoma*.tw,kf. | 1840 | Advanced |  |  |  |
| 68 | germinoma*.tw,kf. | 2222 | Advanced |  |  |  |
| 69 | glioblastom*.tw,kf. | 55090 | Advanced |  |  |  |
| 70 | glioma*.tw,kf. | 77386 | Advanced |  |  |  |
| 71 | gliosarcom*.tw,kf. | 1276 | Advanced |  |  |  |
| 72 | glomus jugulare*.tw,kf. | 908 | Advanced |  |  |  |
| 73 | glomus tympanicum*.tw,kf. | 240 | Advanced |  |  |  |
| 74 | glucagonoma*.tw,kf. | 1016 | Advanced |  |  |  |
| 75 | gonadoblastom*.tw,kf. | 911 | Advanced |  |  |  |
| 76 | GCTOB.tw,kf. | 11 | Advanced |  |  |  |
| 77 | GIST?.tw,kf. | 10368 | Advanced |  |  |  |
| 78 | h?emangioendotheliom*.tw,kf. | 4058 | Advanced |  |  |  |
| 79 | h?emangiom*.tw,kf. | 27315 | Advanced |  |  |  |
| 80 | h?emangiopericytom*.tw,kf. | 3742 | Advanced |  |  |  |
| 81 | h?emangiosarcom*.tw,kf. | 1545 | Advanced |  |  |  |
| 82 | hamartoblastom*.tw,kf. | 49 | Advanced |  |  |  |
| 83 | hepatoblastom*.tw,kf. | 4370 | Advanced |  |  |  |
| 84 | hepatoma*.tw,kf. | 30994 | Advanced |  |  |  |
| 85 | histiocytom*.tw,kf. | 6026 | Advanced |  |  |  |
| 86 | hodgkin*.tw,kf. | 76613 | Advanced |  |  |  |
| 87 | nonhodgkin*.tw,kf. | 139 | Advanced |  |  |  |
| 88 | (hutchinson* adj2 freckle*).tw,kf. | 63 | Advanced |  |  |  |
| 89 | HNPCC.tw,kf. | 2339 | Advanced |  |  |  |
| 90 | immunocytom*.tw,kf. | 619 | Advanced |  |  |  |
| 91 | incidentaloma?.tw,kf. | 3011 | Advanced |  |  |  |
| 92 | insulinoma*.tw,kf. | 7420 | Advanced |  |  |  |
| 93 | kasabach merrit*.tw,kf. | 882 | Advanced |  |  |  |
| 94 | leiomyoblastom*.tw,kf. | 404 | Advanced |  |  |  |
| 95 | leiomyom*.tw,kf. | 17995 | Advanced |  |  |  |
| 96 | leiomyosarcom*.tw,kf. | 12133 | Advanced |  |  |  |
| 97 | leuk?em*.tw,kf. | 317149 | Advanced |  |  |  |
| 98 | preleuk?em*.tw,kf. | 1854 | Advanced |  |  |  |
| 99 | leukoplak*.tw,kf. | 5715 | Advanced |  |  |  |
| 100 | li-fraumeni*.tw,kf. | 1693 | Advanced |  |  |  |
| 101 | lipoblastom*.tw,kf. | 595 | Advanced |  |  |  |
| 102 | lipoma*.tw,kf. | 17424 | Advanced |  |  |  |
| 103 | liposarcom*.tw,kf. | 8268 | Advanced |  |  |  |
| 104 | luteoma*.tw,kf. | 257 | Advanced |  |  |  |
| 105 | lymphangio*.tw,kf. | 15954 | Advanced |  |  |  |
| 106 | lymphoblastom*.tw,kf. | 375 | Advanced |  |  |  |
| 107 | lymphocytom*.tw,kf. | 374 | Advanced |  |  |  |
| 108 | lymphoma*.tw,kf. | 219084 | Advanced |  |  |  |
| 109 | lymphosarcom*.tw,kf. | 5250 | Advanced |  |  |  |
| 110 | lynch*.tw,kf. | 6149 | Advanced |  |  |  |
| 111 | macroglobulinem*.tw,kf. | 4430 | Advanced |  |  |  |
| 112 | m?croprolactinom*.tw,kf. | 974 | Advanced |  |  |  |
| 113 | mastocytom*.tw,kf. | 1924 | Advanced |  |  |  |
| 114 | mastocytos?s*.tw,kf. | 4603 | Advanced |  |  |  |
| 115 | medulloblastom*.tw,kf. | 10194 | Advanced |  |  |  |
| 116 | meigs*.tw,kf. | 966 | Advanced |  |  |  |
| 117 | melanoameloblastom*.tw,kf. | 10 | Advanced |  |  |  |
| 118 | melanoblastom*.tw,kf. | 476 | Advanced |  |  |  |
| 119 | melanocarcin*.tw,kf. | 104 | Advanced |  |  |  |
| 120 | melanoma*.tw,kf. | 148340 | Advanced |  |  |  |
| 121 | melanosis.tw,kf. | 2813 | Advanced |  |  |  |
| 122 | melanotic*.tw,kf. | 2852 | Advanced |  |  |  |
| 123 | meningiom*.tw,kf. | 26288 | Advanced |  |  |  |
| 124 | mesenchymom*.tw,kf. | 871 | Advanced |  |  |  |
| 125 | mesoblast*.tw,kf. | 809 | Advanced |  |  |  |
| 126 | mesonephrom*.tw,kf. | 123 | Advanced |  |  |  |
| 127 | mesotheliom*.tw,kf. | 19573 | Advanced |  |  |  |
| 128 | metaplas*.tw,kf. | 26520 | Advanced |  |  |  |
| 129 | muir-torre*.tw,kf. | 563 | Advanced |  |  |  |
| 130 | myelolipom*.tw,kf. | 1282 | Advanced |  |  |  |
| 131 | myoepitheliom*.tw,kf. | 872 | Advanced |  |  |  |
| 132 | myofibrom*.tw,kf. | 811 | Advanced |  |  |  |
| 133 | myeloma*.tw,kf. | 67500 | Advanced |  |  |  |
| 134 | myoma*.tw,kf. | 7153 | Advanced |  |  |  |
| 135 | myosarcom*.tw,kf. | 239 | Advanced |  |  |  |
| 136 | myxofibrosarcom*.tw,kf. | 831 | Advanced |  |  |  |
| 137 | myxoma*.tw,kf. | 10748 | Advanced |  |  |  |
| 138 | myxosarcom*.tw,kf. | 286 | Advanced |  |  |  |
| 139 | n?evocarcin*.tw,kf. | 78 | Advanced |  |  |  |
| 140 | neurilemmom*.tw,kf. | 2451 | Advanced |  |  |  |
| 141 | neurocytom*.tw,kf. | 891 | Advanced |  |  |  |
| 142 | neuroectodermal*.tw,kf. | 7499 | Advanced |  |  |  |
| 143 | neurofibroma*.tw,kf. | 21515 | Advanced |  |  |  |
| 144 | neurofibrosarcom*.tw,kf. | 431 | Advanced |  |  |  |
| 145 | neurilemmom*.tw,kf. | 2451 | Advanced |  |  |  |
| 146 | neuroblastom*.tw,kf. | 42965 | Advanced |  |  |  |
| 147 | neuroma*.tw,kf. | 11724 | Advanced |  |  |  |
| 148 | neurothekeom*.tw,kf. | 286 | Advanced |  |  |  |
| 149 | NSCLC.tw,kf. | 67448 | Advanced |  |  |  |
| 150 | odontoma*.tw,kf. | 1687 | Advanced |  |  |  |
| 151 | oligo*.tw,kf. | 314977 | Advanced |  |  |  |
| 152 | osteoblastom*.tw,kf. | 1343 | Advanced |  |  |  |
| 153 | osteochondrom*.tw,kf. | 3731 | Advanced |  |  |  |
| 154 | osteoclastom*.tw,kf. | 395 | Advanced |  |  |  |
| 155 | osteoma*.tw,kf. | 12587 | Advanced |  |  |  |
| 156 | osteosarcom*.tw,kf. | 31096 | Advanced |  |  |  |
| 157 | papilloma*.tw,kf. | 74221 | Advanced |  |  |  |
| 158 | papillary*.tw,kf. | 69839 | Advanced |  |  |  |
| 159 | paragangliom*.tw,kf. | 9478 | Advanced |  |  |  |
| 160 | pheochromocytom*.tw,kf. | 20251 | Advanced |  |  |  |
| 161 | phyllo?des*.tw,kf. | 2803 | Advanced |  |  |  |
| 162 | pinealocytoma*.tw,kf. | 18 | Advanced |  |  |  |
| 163 | pinealoma*.tw,kf. | 366 | Advanced |  |  |  |
| 164 | pineoblastoma*.tw,kf. | 530 | Advanced |  |  |  |
| 165 | pineocytoma*.tw,kf. | 295 | Advanced |  |  |  |
| 166 | plasmacytom*.tw,kf. | 7540 | Advanced |  |  |  |
| 167 | (polycythem* adj2 vera?).tw,kf. | 6697 | Advanced |  |  |  |
| 168 | prolactinom*.tw,kf. | 4012 | Advanced |  |  |  |
| 169 | retinoblastom*.tw,kf. | 18727 | Advanced |  |  |  |
| 170 | rhabdoid*.tw,kf. | 3602 | Advanced |  |  |  |
| 171 | rhabdomyom*.tw,kf. | 1812 | Advanced |  |  |  |
| 172 | rhabdomyosarcom*.tw,kf. | 14118 | Advanced |  |  |  |
| 173 | sarcom*.tw,kf. | 131690 | Advanced |  |  |  |
| 174 | seminoma*.tw,kf. | 8288 | Advanced |  |  |  |
| 175 | Sertoli- Leydig.tw,kf. | 792 | Advanced |  |  |  |
| 176 | somatostatinoma*.tw,kf. | 406 | Advanced |  |  |  |
| 177 | somatotrophinom*.tw,kf. | 89 | Advanced |  |  |  |
| 178 | struma ovarii*.tw,kf. | 709 | Advanced |  |  |  |
| 179 | thecoma*.tw,kf. | 499 | Advanced |  |  |  |
| 180 | teratocarcin*.tw,kf. | 2795 | Advanced |  |  |  |
| 181 | teratoma*.tw,kf. | 17719 | Advanced |  |  |  |
| 182 | thymom*.tw,kf. | 11233 | Advanced |  |  |  |
| 183 | trophoblast*.tw,kf. | 27248 | Advanced |  |  |  |
| 184 | vipoma*.tw,kf. | 431 | Advanced |  |  |  |
| 185 | wilms*.tw,kf. | 11471 | Advanced |  |  |  |
| 186 | or/1-185 | 6060735 | Advanced |  |  |  |
| 187 | Family/ | 86730 | Advanced |  |  |  |
| 188 | Family Relations/ | 12228 | Advanced |  |  |  |
| 189 | Family Support/ | 157 | Advanced |  |  |  |
| 190 | Intergenerational Relations/ | 4555 | Advanced |  |  |  |
| 191 | Maternal Behavior/ | 12274 | Advanced |  |  |  |
| 192 | Parent-Child Relations/ | 37250 | Advanced |  |  |  |
| 193 | Father-Child Relations/ | 3915 | Advanced |  |  |  |
| 194 | Mother-Child Relations/ | 22422 | Advanced |  |  |  |
| 195 | Parenting/ | 22634 | Advanced |  |  |  |
| 196 | Paternal Behavior/ | 1956 | Advanced |  |  |  |
| 197 | Grandparents/ | 823 | Advanced |  |  |  |
| 198 | Parents/ | 86432 | Advanced |  |  |  |
| 199 | Fathers/ | 11455 | Advanced |  |  |  |
| 200 | Mothers/ | 59748 | Advanced |  |  |  |
| 201 | Single Parent/ | 1250 | Advanced |  |  |  |
| 202 | Caregivers/ | 54806 | Advanced |  |  |  |
| 203 | ((intergeneration* or inter-generation*) adj3 relation*).tw,kf. | 946 | Advanced |  |  |  |
| 204 | ((family or families or parent*) adj3 relation*).tw,kf. | 35643 | Advanced |  |  |  |
| 205 | ((father? or mother?) adj3 relation*).tw,kf. | 6939 | Advanced |  |  |  |
| 206 | ((grandparent? or grandfather? or grandmother?) adj3 relation*).tw,kf. | 252 | Advanced |  |  |  |
| 207 | ((caregiv* or care-giv* or carer?) adj3 relation*).tw,kf. | 4085 | Advanced |  |  |  |
| 208 | ((family or families or parent*) adj3 accommodat*).tw,kf. | 692 | Advanced |  |  |  |
| 209 | ((father? or mother?) adj3 accommodat*).tw,kf. | 65 | Advanced |  |  |  |
| 210 | ((grandparent? or grandfather? or grandmother?) adj3 accommodat*).tw,kf. | 1 | Advanced |  |  |  |
| 211 | ((caregiv* or care-giv* or carer?) adj3 accommodat*).tw,kf. | 89 | Advanced |  |  |  |
| 212 | ((family or families or parent*) adj3 behavio?r*).tw,kf. | 21369 | Advanced |  |  |  |
| 213 | ((father? or mother?) adj3 behavio?r*).tw,kf. | 4176 | Advanced |  |  |  |
| 214 | ((grandparent? or grandfather? or grandmother?) adj3 behavio?r*).tw,kf. | 62 | Advanced |  |  |  |
| 215 | ((caregiv* or care-giv* or carer?) adj3 behavio?r*).tw,kf. | 2960 | Advanced |  |  |  |
| 216 | ((family or families or parent*) adj3 capacit*).tw,kf. | 1292 | Advanced |  |  |  |
| 217 | ((father? or mother?) adj3 capacit*).tw,kf. | 231 | Advanced |  |  |  |
| 218 | ((grandparent? or grandfather? or grandmother?) adj3 capacit*).tw,kf. | 8 | Advanced |  |  |  |
| 219 | ((caregiv* or care-giv* or carer?) adj3 capacit*).tw,kf. | 323 | Advanced |  |  |  |
| 220 | ((family or families or parent*) adj3 (comfort* or overcomfort*)).tw,kf. | 824 | Advanced |  |  |  |
| 221 | ((father? or mother?) adj3 (comfort* or overcomfort*)).tw,kf. | 135 | Advanced |  |  |  |
| 222 | ((grandparent? or grandfather? or grandmother?) adj3 (comfort* or overcomfort*)).tw,kf. | 0 | Advanced |  |  |  |
| 223 | ((caregiv* or care-giv* or carer?) adj3 (comfort* or overcomfort*)).tw,kf. | 208 | Advanced |  |  |  |
| 224 | ((family or families or parent*) adj3 (control* or overcontrol*)).tw,kf. | 15444 | Advanced |  |  |  |
| 225 | ((father? or mother?) adj3 (control* or overcontrol*)).tw,kf. | 5659 | Advanced |  |  |  |
| 226 | ((grandparent? or grandfather? or grandmother?) adj3 (control* or overcontrol*)).tw,kf. | 40 | Advanced |  |  |  |
| 227 | ((caregiv* or care-giv* or carer?) adj3 (control* or overcontrol*)).tw,kf. | 1403 | Advanced |  |  |  |
| 228 | ((family or families or parent*) adj3 (engag* or overengag*)).tw,kf. | 6329 | Advanced |  |  |  |
| 229 | ((father? or mother?) adj3 (engag* or overengag*)).tw,kf. | 1152 | Advanced |  |  |  |
| 230 | ((grandparent? or grandfather? or grandmother?) adj3 (engag* or overengag*)).tw,kf. | 32 | Advanced |  |  |  |
| 231 | ((caregiv* or care-giv* or carer?) adj3 (engag* or overengag*)).tw,kf. | 1451 | Advanced |  |  |  |
| 232 | enmesh*.tw,kf. | 1042 | Advanced |  |  |  |
| 233 | ((family or families or parent*) adj3 helicopter*).tw,kf. | 72 | Advanced |  |  |  |
| 234 | ((father? or mother?) adj3 helicopter*).tw,kf. | 7 | Advanced |  |  |  |
| 235 | ((grandparent? or grandfather? or grandmother?) adj3 helicopter*).tw,kf. | 0 | Advanced |  |  |  |
| 236 | ((caregiv* or care-giv* or carer?) adj3 helicopter*).tw,kf. | 0 | Advanced |  |  |  |
| 237 | ((family or families or parent*) adj3 (involv* or overinvolv*)).tw,kf. | 23582 | Advanced |  |  |  |
| 238 | ((father? or mother?) adj3 (involv* or overinvolv*)).tw,kf. | 2701 | Advanced |  |  |  |
| 239 | ((grandparent? or grandfather? or grandmother?) adj3 (involv* or overinvolv*)).tw,kf. | 144 | Advanced |  |  |  |
| 240 | ((caregiv* or care-giv* or carer?) adj3 (involv* or overinvolv*)).tw,kf. | 3056 | Advanced |  |  |  |
| 241 | ((family or families or parent*) adj3 intens*).tw,kf. | 2296 | Advanced |  |  |  |
| 242 | ((father? or mother?) adj3 intens*).tw,kf. | 356 | Advanced |  |  |  |
| 243 | ((grandparent? or grandfather? or grandmother?) adj3 intens*).tw,kf. | 18 | Advanced |  |  |  |
| 244 | ((caregiv* or care-giv* or carer?) adj3 intens*).tw,kf. | 556 | Advanced |  |  |  |
| 245 | ((family or families or parent*) adj3 intrusive*).tw,kf. | 199 | Advanced |  |  |  |
| 246 | ((father? or mother?) adj3 intrusive*).tw,kf. | 92 | Advanced |  |  |  |
| 247 | ((grandparent? or grandfather? or grandmother?) adj3 intrusive*).tw,kf. | 0 | Advanced |  |  |  |
| 248 | ((caregiv* or care-giv* or carer?) adj3 intrusive*).tw,kf. | 23 | Advanced |  |  |  |
| 249 | ((family or families or parent*) adj3 (micromanag* or micro-manag*)).tw,kf. | 2 | Advanced |  |  |  |
| 250 | ((father? or mother?) adj3 (micromanag* or micro-manag*)).tw,kf. | 0 | Advanced |  |  |  |
| 251 | ((grandparent? or grandfather? or grandmother?) adj3 (micromanag* or micro-manag*)).tw,kf. | 0 | Advanced |  |  |  |
| 252 | ((caregiv* or care-giv* or carer?) adj3 (micromanag* or micro-manag*)).tw,kf. | 0 | Advanced |  |  |  |
| 253 | (paternali?m* or overparent*).tw,kf. | 1639 | Advanced |  |  |  |
| 254 | ((family or families or parent*) adj3 (practic* or practis*)).tw,kf. | 22422 | Advanced |  |  |  |
| 255 | ((father? or mother?) adj3 (practic* or practis*)).tw,kf. | 2494 | Advanced |  |  |  |
| 256 | ((grandparent? or grandfather? or grandmother?) adj3 (practic* or practis*)).tw,kf. | 55 | Advanced |  |  |  |
| 257 | ((caregiv* or care-giv* or carer?) adj3 (practic* or practis*)).tw,kf. | 1668 | Advanced |  |  |  |
| 258 | ((family or families or parent*) adj3 (protect* or overprotect*)).tw,kf. | 4347 | Advanced |  |  |  |
| 259 | ((father? or mother?) adj3 (protect* or overprotect*)).tw,kf. | 1447 | Advanced |  |  |  |
| 260 | ((grandparent? or grandfather? or grandmother?) adj3 (protect* or overprotect*)).tw,kf. | 25 | Advanced |  |  |  |
| 261 | ((caregiv* or care-giv* or carer?) adj3 (protect* or overprotect*)).tw,kf. | 357 | Advanced |  |  |  |
| 262 | ((family or families or parent*) adj3 (reliant* or overreliant*)).tw,kf. | 62 | Advanced |  |  |  |
| 263 | ((father? or mother?) adj3 (reliant* or overreliant*)).tw,kf. | 6 | Advanced |  |  |  |
| 264 | ((grandparent? or grandfather? or grandmother?) adj3 (reliant* or overreliant*)).tw,kf. | 0 | Advanced |  |  |  |
| 265 | ((caregiv* or care-giv* or carer?) adj3 (reliant* or overreliant*)).tw,kf. | 30 | Advanced |  |  |  |
| 266 | ((family or families or parent*) adj3 routine?).tw,kf. | 1528 | Advanced |  |  |  |
| 267 | ((father? or mother?) adj3 routine?).tw,kf. | 254 | Advanced |  |  |  |
| 268 | ((grandparent? or grandfather? or grandmother?) adj3 routine?).tw,kf. | 1 | Advanced |  |  |  |
| 269 | ((caregiv* or care-giv* or carer?) adj3 routine?).tw,kf. | 295 | Advanced |  |  |  |
| 270 | ((family or families or parent*) adj3 rule?).tw,kf. | 883 | Advanced |  |  |  |
| 271 | ((father? or mother?) adj3 rule?).tw,kf. | 70 | Advanced |  |  |  |
| 272 | ((grandparent? or grandfather? or grandmother?) adj3 rule?).tw,kf. | 6 | Advanced |  |  |  |
| 273 | ((caregiv* or care-giv* or carer?) adj3 rule?).tw,kf. | 35 | Advanced |  |  |  |
| 274 | ((family or families or parent*) adj3 (shelter* or overshelter*)).tw,kf. | 236 | Advanced |  |  |  |
| 275 | ((father? or mother?) adj3 (shelter* or overshelter*)).tw,kf. | 53 | Advanced |  |  |  |
| 276 | ((grandparent? or grandfather? or grandmother?) adj3 (shelter* or overshelter*)).tw,kf. | 1 | Advanced |  |  |  |
| 277 | ((caregiv* or care-giv* or carer?) adj3 (shelter* or overshelter*)).tw,kf. | 20 | Advanced |  |  |  |
| 278 | ((family or families or parent*) adj3 smother*).tw,kf. | 4 | Advanced |  |  |  |
| 279 | ((father? or mother?) adj3 smother*).tw,kf. | 6 | Advanced |  |  |  |
| 280 | ((grandparent? or grandfather? or grandmother?) adj3 smother*).tw,kf. | 0 | Advanced |  |  |  |
| 281 | ((caregiv* or care-giv* or carer?) adj3 smother*).tw,kf. | 2 | Advanced |  |  |  |
| 282 | ((family or families or parent*) adj3 (snowplow* or snow-plow*)).tw,kf. | 1 | Advanced |  |  |  |
| 283 | ((father? or mother?) adj3 (snowplow* or snow-plow*)).tw,kf. | 0 | Advanced |  |  |  |
| 284 | ((grandparent? or grandfather? or grandmother?) adj3 (snowplow* or snow-plow*)).tw,kf. | 0 | Advanced |  |  |  |
| 285 | ((caregiv* or care-giv* or carer?) adj3 (snowplow* or snow-plow*)).tw,kf. | 0 | Advanced |  |  |  |
| 286 | ((family or families or parent*) adj3 strateg*).tw,kf. | 6619 | Advanced |  |  |  |
| 287 | ((father? or mother?) adj3 strateg*).tw,kf. | 835 | Advanced |  |  |  |
| 288 | ((grandparent? or grandfather? or grandmother?) adj3 strateg*).tw,kf. | 18 | Advanced |  |  |  |
| 289 | ((caregiv* or care-giv* or carer) adj3 strateg*).tw,kf. | 1156 | Advanced |  |  |  |
| 290 | ((family or families or parent*) adj3 (supervi* or oversupervi*)).tw,kf. | 1505 | Advanced |  |  |  |
| 291 | ((father? or mother?) adj3 (supervi* or oversupervi*)).tw,kf. | 116 | Advanced |  |  |  |
| 292 | ((grandparent? or grandfather? or grandmother?) adj3 (supervi* or oversupervi*)).tw,kf. | 6 | Advanced |  |  |  |
| 293 | ((caregiv* or care-giv* or carer?) adj3 (supervi* or oversupervi*)).tw,kf. | 253 | Advanced |  |  |  |
| 294 | ((family or families or parent*) adj3 (support* or oversupport*)).tw,kf. | 41289 | Advanced |  |  |  |
| 295 | ((father? or mother?) adj3 (support* or oversupport*)).tw,kf. | 4891 | Advanced |  |  |  |
| 296 | ((grandparent? or grandfather? or grandmother) adj3 (support* or oversupport*)).tw,kf. | 155 | Advanced |  |  |  |
| 297 | ((caregiv* or care-giv* or carer) adj3 (support* or oversupport*)).tw,kf. | 8246 | Advanced |  |  |  |
| 298 | ((family or families or parent*) adj3 tenden*).tw,kf. | 380 | Advanced |  |  |  |
| 299 | ((father? or mother?) adj3 tenden*).tw,kf. | 162 | Advanced |  |  |  |
| 300 | ((grandparent? or grandfather? or grandmother) adj3 tenden*).tw,kf. | 0 | Advanced |  |  |  |
| 301 | ((caregiv* or care-giv* or carer?) adj3 tenden*).tw,kf. | 38 | Advanced |  |  |  |
| 302 | or/187-301 | 454440 | Advanced |  |  |  |
| 303 | (child* or preschool or pre school or young or young adult? or adolescen* or teenage* or teen-age* or p?ediatric* or AYA or TYA or TYACS* or AYACS*).mp,kw. | 4974115 | Advanced |  |  |  |
| 304 | 186 and 302 and 303 | 13419 | Advanced |  |  |  |
| 305 | limit 304 to "humans only (removes records about animals)" | 13402 | Advanced |  |  |  |
| 306 | limit 305 to (clinical conference or clinical trial, veterinary or clinical trial protocol or consensus development conference or consensus development conference, nih or news or newspaper article or preprint or retracted publication or "retraction of publication") | 78 | Advanced |  |  |  |
| 307 | 305 not 306 | 13324 | Advanced |  |  |  |
| 308 | limit 307 to english language | 12517 | Advanced |  |  |  |
|  | | |  | |  | |
